# Nanoparticle “switch-on” by tetrazine triggering†
†Electronic supplementary information (ESI) available. See DOI: 10.1039/c6cc05118a



**DOI:** 10.1039/c6cc05118a

**Published:** 2016-08-19

**Authors:** Kevin Neumann, Sarthak Jain, Jin Geng, Mark Bradley

**Affiliations:** a School of Chemistry , University of Edinburgh , Joseph Black Building , Edinburgh , EH9 3FJ , UK . Email: jin.geng@ed.ac.uk ; Email: mark.bradley@ed.ac.uk

## Abstract

This work describes how a small-molecule chemical trigger, reacting through the mediatory of an inverse electron demand Diels–Alder reaction, results in enhanced cellular uptake and selective nanoparticle disintegration and cargo liberation, *via* gross polymeric morphological alterations. The power of these responsive nanoparticles is demonstrated through encapsulation of the anti-cancer agent doxorubicin and its triggered release, allowing controlled cell death in response to a small-molecule chemical trigger.

## Abstract 2

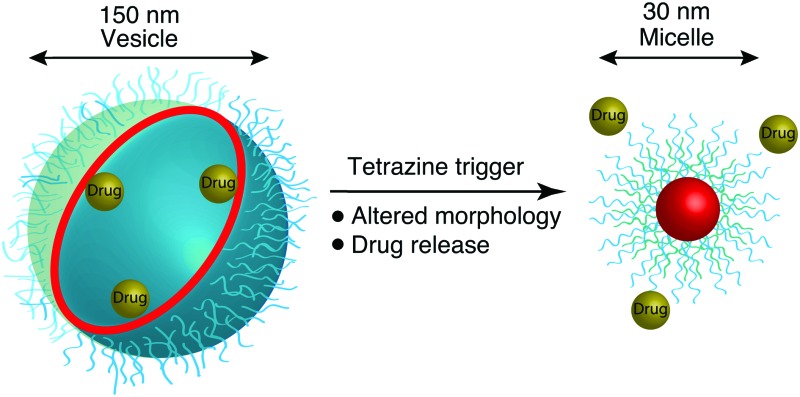
Small-molecule mediated release of drugs from self-assembled nanoparticles through the mediatory of a Diels–Alder reaction.

Nanotechnology-based systems for drug delivery have received tremendous attention and delivered impressive and symbiotic progress in materials science and pharmaceutical development.^1–3^ Nanoparticles (NP), have been used to improve drug solubility and enhance therapeutic effectiveness,^4,5^ owing to targeting to tumor tissues with improved pharmacokinetics and pharmacodynamics and active intracellular delivery. In this context nanoparticle-based polymersomes, generated from polymeric amphiphiles, are popular allowing the rapid generation of stable vesicles,^6–8^ and micelles^9–11^ in water. Typically, the membranes of polymersomes are thicker, stronger, and inherently more stable than those found in conventional liposomes, while the scope of polymeric building blocks available for membrane formation allows for much greater chemical control and tunability then conventional lipid nano-structures.^12,13^ Typically micelles encapsulate hydrophobic compounds, while vesicles can encapsulate hydrophilic molecules within their aqueous interior as well as trapping hydrophobic moieties within the “membrane” with drugs such as doxorubicin well retained.^14^ Polyethylene glycol (PEG) has been widely used as the hydrophilic block in polymersomes, conferring properties to block immunological recognition, and enhance biocompatibility.^15^

Triggered-release nanoparticles offer a sophisticated approach to drug-delivery enabling control over when and where the drug is released, enhancing therapeutic efficacy and minimising side-effects.^16,17^ In general, triggering causes changes in the hydrophilic-to-hydrophobic balance of the polymer, thereby resulting in morphological disturbance of the self-assembled polymersome structure, and nanoparticle conformational instability.^18^

Controlled release from nanoparticles through the application of an external stimulus can be broadly divided into remote and local triggers. Remote triggers use an external physical stimulus such as temperature (*e.g.* polymers exhibiting a lower critical solution temperature, for example, poly(*N*-isopropylacrylamide)),^19^ ultrasound or light (which can alter the properties of the nanoparticles with the help of a molecular switch such as an azobenzene unit^20^). Local triggers utilise the environment of the target site, for example, an up-regulation in a specific enzyme (*e.g.* a protease), a change in pH, or reactive oxygen species (often associated with tumors). As such reactive oxygen species, such as hydrogen peroxide and hydroxyl radicals, have been employed as triggers to create oxidation sensitive drug delivery systems based on thioether^21^ or selenium oxidation.^22^ In addition, drugs have been released from polymers through cleavage of disulphide linkages by glutathione (GSH)^23,24^ or dithiothreitol (DTT)^25,26^ in the form of degradable polymer aggregates.

A powerful reaction that has recently been widely exploited in bioconjugation^27,28^ strategies is the additive free, inverse electron demand Diels–Alder reaction, between tetrazines and electron rich dienophiles,^29^ while, the inverse electron demand Diels–Alder reaction in an aqueous environment displays an acceleration behaviour as previous reported by both us and others.^30,31^ Here we report the development of a fully bioorthogonal, small molecule activated nanoparticle, with on-demand drug release. Using a small molecule, in the form of a tetrazine as an external trigger, polymer chains that make up the nanoparticles undergo a series of inverse electron demand Diels–Alder reactions, disrupting the nanoparticle, with concomitant release of an encapsulated drug – in essence we demonstrate tetrazine responsive nanoparticles with “switch-on” release of the anti-cancer agent doxorubicin. PC3 human prostate cancer cell lines were chosen for investigating their response to doxorubicin loaded nanoparticles.

Poly(allyl glycidyl ethers) (PAGE) have been explored as a chemically flexible alternative to PEG, stemming from the pendant allyl groups which are amenable to a range of modifications. In this study, a diblock copolymer, poly(ethylene glycol)-*b*-(allyl glycidyl ether) (**PEG-*b*-PAGE**), was synthesised *via* anionic ring-opening polymerisation using potassium alkoxide/naphthalenide as the initiator^32^ to give a block copolymer that contained a hydrophilic PEG block with a weight fraction of 30% and a hydrophobic block with terminal allyl pendant units. This was formulated to form compartmental self-assembled nanoparticles (Fig. 1), with transmission electron microscopy (TEM) analysis revealing the formation of characteristic hollow nanoparticles with a well-defined spherical morphology, exhibiting a mono-modal distribution of particles of *ca.* 150 ± 32 nm, with a wall thickness estimated to be 4 ± 1 nm (Fig. 1c).

**Fig. 1:**
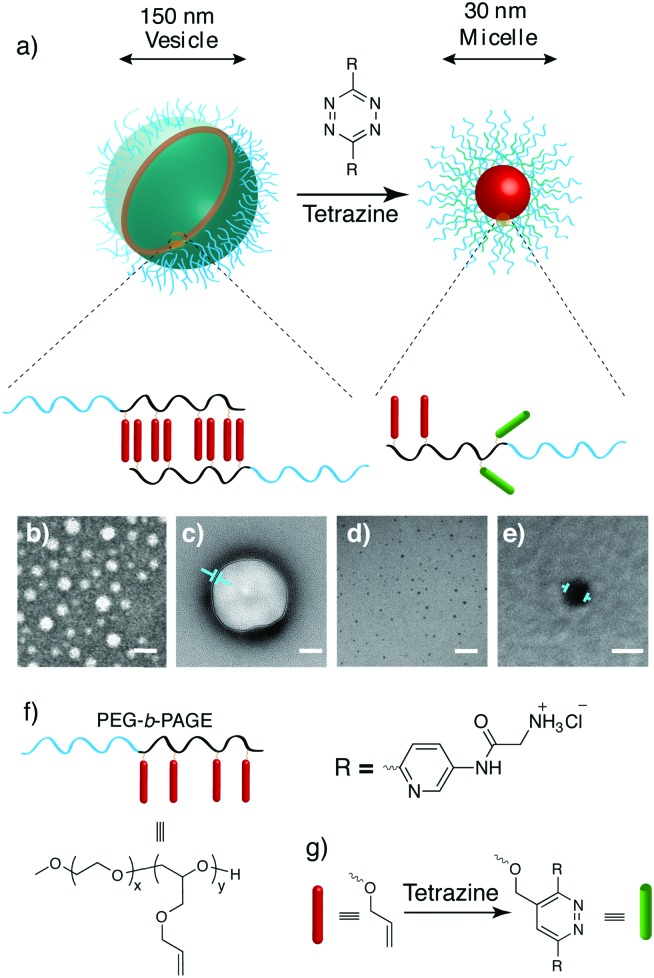
(a) The block co-polymer **PEG-*b*-PAGE** (Mn = 5.8 kDa; PDI = 1.03) forms core–shell nanoarchitectures in water. Modification of the allyl ether groups on the polymer backbone with a tetrazine carrying ammonium groups through an inverse electron demand Diels–Alder reaction resulted in the generation of NP micelles much reduced in size compared to the original vesicles. The morphologies were confirmed by TEM (staining with uranyl acetate) showing in (b) vesicles in the absence of tetrazine. (c) Shows a single vesicle with the membrane thickness indicated by arrows (∼4 nm). (d) Micelles in the presence of tetrazine. (e) Shows a single micelle with diameter ≈30 nm. Scale bars represent 200 nm for (b) and (d), 50 nm for (c) and (e). (f) The chemical structure of the **PEG-*b*-PAGE** used in this study. (g) The reaction of the allyl ether with the tetrazine.

Since the tetrazine is a reactive species, it is important to keep the balance of solution stability and fast reaction kinetics.^33^ We have previous reported that the tetrazine used in this study undergoes a fast reaction with poly(allyl glycidyl ethers) in water.^30^ Upon treatment of the nanoparticles with a water-soluble and hydrophilic tetrazine, the polymer backbone was rapidly modified, with dynamic light scattering studies revealing a dramatic reduction in diameter from 150 nm to 30 nm after four hours incubation (interestingly this displayed a lag phase of 2 hours, see Fig. 2a). The reaction will be slow initially since the hydrophilic tetrazine has to reach to the hydrophobic layers, but following the modification, the rate will increase, as the membrane becomes more and more hydrophilic. TEM measurement confirmed that a population of micelles was generated with a uniform diameter of 30 ± 5 nm (Fig. 1d and e). These changes take place due to the modification of the hydrophobic moieties, arising from the conversion of the hydrophobic moieties into hydrophilic side chains (due to the nature of the tetrazine used and the high level of polymer modification). In support of these observation, the zeta-potential values of the NP exhibited a dramatic change from 0.12 mV to 24.10 mV following tetrazine modification.

**Fig. 2:**
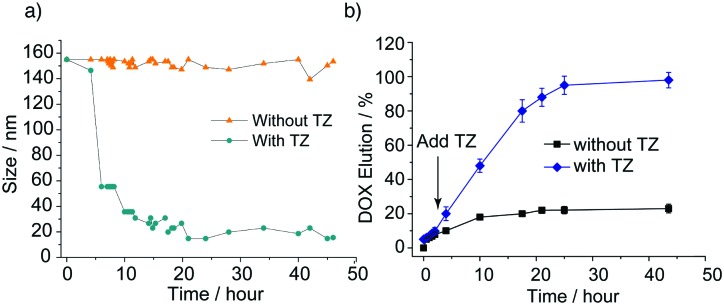
(a) Size change and (b) release profiles with and without tetrazine triggering of vesicles. Error bars represent standard deviations (*n* = 3).

The IC_50_ values of the NPs and tetrazine against the PC3 cells were determined as 5 mg ml^–1^ and 100 μM respectively, indicating relatively low cytotoxicity. To investigate cell internalisation, FITC labelled **PEG-*b*-PAGE** (**FITC-PEG-*b*-PAGE**) was prepared. The self-assembled NPs showed a time-dependent increase in fluorescence intensity with cells with an enhanced internalisation capacity in the presence of tetrazine as evidenced by flow cytometry and cell imaging (Fig. 3).

**Fig. 3:**
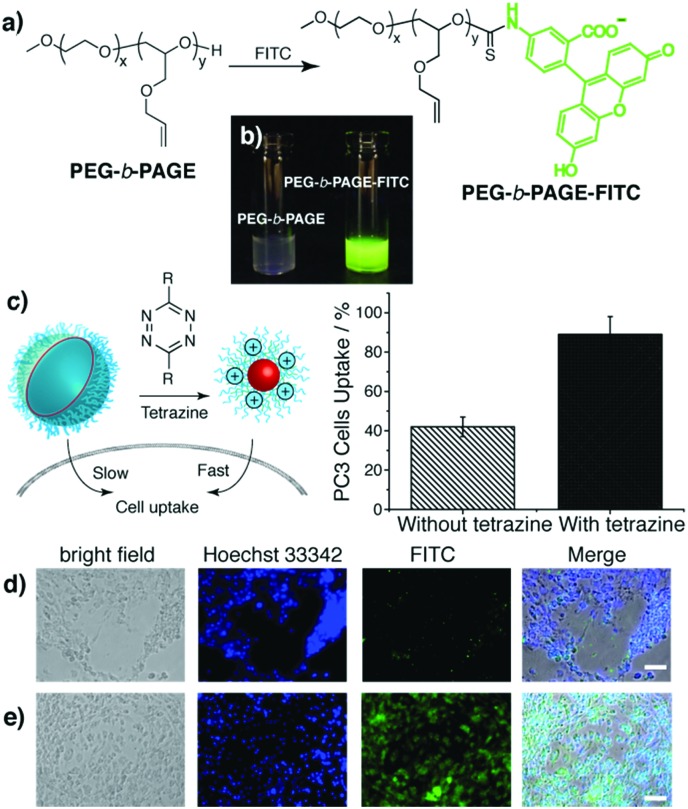
(a) Synthesis of fluorescein labelled block copolymer (**PEG-*b*-PAGE-FITC**) *via* the reaction between fluorescein isothiocyanate (FITC) and the hydroxyl group of the **PEG-*b*-PAGE**. (b) Images of nanoparticles of **PEG-*b*-PAGE** and **PEG-*b*-PAGE-FITC** (both 3 mg ml^–1^) in water upon illumination (350 nm). (c) PC3 cell uptake of **PEG-*b*-PAGE-FITC** NPs after incubation at 37 °C for 72 hours without and with tetrazine (50 μM), as quantified by flow cytometry. (d and e) Images of PC3 cells incubated with **PEG-*b*-PAGE-FITC** NPs at 37 °C for 72 hours; (d) without tetrazine; (e) with tetrazine. Imaging channels: FITC channel (*λ*_ex_ = 470 nm; *λ*_em_ = 525 nm). Green fluorescence is due to **PEG-*b*-PAGE-FITC** NP uptake; cell nuclei were stained with Hoechst 33342 (*λ*_ex_ = 358 nm; *λ*_em_ = 461 nm). Scale bar = 50 μm.

To explore the cargo release profile of the nanoparticles upon the addition of tetrazine, nanoparticles loaded with doxorubicin (DOX) were prepared (the DOX loading efficiency and dimensions of the DOX-loaded NPs are shown in Table 1). For measurement of release profiles, DOX loaded NPs (3 mg ml^–1^ in PBS) were dialysed (MWCO 20 kDa) against PBS (pH = 7.4, 10 mM) at 37 °C and tetrazine (50 μM, 4 equiv. to allyl ether units) was introduced and the release of doxorubicin was monitored spectrophotometrically at 485 nm. The triggered nanoparticles exhibited full cargo release, with the release profile mirroring the reduction in particle diameter (see Fig. 2b). In the absence of tetrazine, the amount of liberated DOX was negligible over 48 h, while in the in the presence of tetrazine, the release was rapid, with almost quantitative release over 10 hours. The trigger release of DOX was attributed to the gross morphological changes occurring to the polymer. Hydrophobic DOX is encapsulated in the membrane of the vesicles. The reaction of tetrazine with the allyl group will cause vesicles collapse rapidly as the global properties change quickly, but small hydrophobic pockets were still binding DOX release.

**Table 1:** Characterisation of the prepared DOX-loaded nanoparticles

DOX loading conc. (μM)	*d* (nm)	PDI	Encapsulation efficiency (%)
5	150 ± 24	0.112	22 ± 4.1
20	145 ± 21	0.107	32 ± 3.6
50	160 ± 36	0.098	35 ± 6.2
100	178 ± 39	0.126	30 ± 4.1

Cell viability studies were undertaken in the presence of DOX loaded NPs, with and without the tetrazine trigger using PC3 cells stained with the vital cell stain CellTracker™ green. Fig. 4 shows the images of cells incubated with empty NPs and NPs loaded with DOX with and without the tetrazine (videos were created through continuous image captures under microscope, see ESI†). After 72 hours cell viability was quantified by flow cytometry using propidium iodide as a live/dead cell discriminator. The proportions of viable and non-viable cells were also evaluated as shown in Fig. 4 with DOX loaded NPs by flow cytometry. 1 μM and 6 μM DOX-NPs were non-cytotoxic over 72 hours in the absence of tetrazine, while the 18 μM DOX-NPs exhibited some cytotoxicity (87% cell viability compared to 95% for the control). These DOX loaded nanoparticles demonstrated significant effects on the cells following the introduction of the tetrazine, which triggered nanoparticle collapse and DOX release, resulting in cell death in a dose dependent manner. As shown in Fig. 4, upon the addition of a trigger tetrazine, a cytotoxic effect was observed for all concentrations with the 18 μM DOX loaded NP's resulting in 98% of cells taking up the stain propidium iodide (which is excluded by the plasma membrane in healthy cells) – an indication of cell death. In addition to triggering cargo release the tetrazine reaction also promoted cellular uptake, resulting in DOX-loaded nanoparticles increasingly entering cells as the reaction progressed.^34,35^

**Fig. 4:**
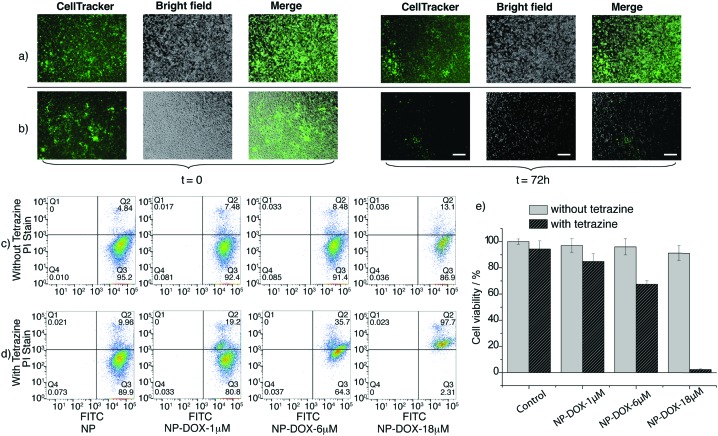
PC3 cells were stained with CellTracker™ Green prior to incubation with NPs which were loaded with DOX (at 1 μM, 6 μM and 18 μM) at 37 °C in the presence of 5% CO_2_ for 72 hours. (a and b) Microscopy images of PC3 cells incubated with (a) empty NPs and (b) NPs loaded with 18 μm DOX in the presence of tetrazine (50 μM) at *t* = 0 and *t* = 72 hours. (c and d) Cells were harvested, stained with propidium iodide PI (1 μg ml^–1^) and examined by flow cytometry. The *x*-axis measures the fluorescein intensity of cells (FITC) due to CellTracker™ staining, (cell viability) and the *y*-axis corresponds to the PI fluorescence intensity due to the dead cell staining. Forward *versus* Side scatter profiles were used to gate-in intact cellular materials (remove cell debris) and determine the live cells and dead cells of each cell (see ESI†). Quadrant regions were derived from the gate and set to delineate the FITC and PI population. (c) Without and (d) with tetrazine triggering for different concentration of DOX loaded NPs. (e) Summary of cell viability in the presence of different concentration of DOX loaded NPs (errors are the standard deviation from the means, *n* = 3). Scale bar = 200 μM.

In summary, a novel approach to allow the selective control and manipulation and subsequent triggering of nanoparticles by the application of an external bio-orthogonal chemical stimulus in the form of a tetrazine is presented. The tetrazine mediated inverse electron demand Diels Alder reaction alters the morphology of the nanoparticles, triggering drug release. With a tetrazine trigger, the NPs also exhibited enhanced cell uptake due to the switching of the surface charge and a reduction in size. This new class of responsive material offers a new control strategy for triggered release through a chemical stimulus using a tetrazine, with potential in dosage control. There are a number of strategies for controlled drug release that have been designed to increase intracellular drug concentrations, but efficient delivery of therapeutics into tumour cells still remains a major challenge for cancer therapy. Recently, tailor-made dual-responsive drug delivery devices have been designed to overcome drug resistance and inefficient cellular uptake.^36,37^ Our approach offers a new strategy in the manipulation of polymeric nanoparticles; gaining control over both size and morphology of the self-assembled structures. With chemical handles, our design can be simply employed in the creation of robust multiple-responsive delivery devices by the combination of a tetrazine trigger with, for example, pH sensitivity, potentially providing a novel and versatile approach for efficient cancer therapy.

This work was supported by the European Research Council (Advanced Grant ADREEM ERC-2013-340469) and Biotechnology and Biological Sciences Research Council (BB/L00609X/1). We thank Dr M. Waterfall for flow cytometry analysis.

## References

[cit1] Mura S., Nicolas J., Couvreur P. (2013). Nat. Mater..

[cit2] De M., Ghosh P. S., Rotello V. M. (2008). Adv. Mater..

[cit3] Cabral H., Nishiyama N., Kataoka K. (2011). Acc. Chem. Res..

[cit4] Shapira A., Livney Y. D., Broxterman H. J., Assaraf Y. G. (2011). Drug Resist. Updates.

[cit5] Venkataraman S., Hedrick J. L., Ong Z. Y., Yang C., Ee P. L. R., Hammond P. T., Yang Y. Y. (2011). Adv. Drug Delivery Rev..

[cit6] Discher D. (2002). Science.

[cit7] Savić R., Luo L., Eisenberg A., Maysinger D. (2003). Science.

[cit8] Allen C., Maysinger D., Eisenberg A. (1999). Colloids Surf., B.

[cit9] Zhang Q., Ko N. R., Oh J. K. (2012). Chem. Commun..

[cit10] Kataoka K., Harada A., Nagasaki Y. (2001). Adv. Drug Delivery Rev..

[cit11] Blanazs A., Armes S. P., Ryan A. J. (2009). Macromol. Rapid Commun..

[cit12] Pattni B. S., Chupin V. V., Torchilin V. P. (2015). Chem. Rev..

[cit13] Elsabahy M., Wooley K. L. (2012). Chem. Soc. Rev..

[cit14] Meng F., Zhong Z., Feijen J. (2009). Biomacromolecules.

[cit15] Pasut G., Veronese F. M. (2009). Adv. Drug Delivery Rev..

[cit16] Esser-Kahn A. P., Odom S. A., Sottos N. R., White S. R., Moore J. S. (2011). Macromolecules.

[cit17] Fleige E., Quadir M. A., Haag R. (2012). Adv. Drug Delivery Rev..

[cit18] Stuart M. A. C., Huck W. T. S., Genzer J., Müller M., Ober C., Stamm M., Sukhorukov G. B., Szleifer I., Tsukruk V. V., Urban M., Winnik F., Zauscher S., Luzinov I., Minko S. (2010). Nat. Mater..

[cit19] Schild H. G. (1992). Prog. Polym. Sci..

[cit20] Wang G., Xia Tong A., Zhao Y. (2004). Macromolecules.

[cit21] Napoli A., Valentini M., Tirelli N., Müller M. (2004). Nat. Mater..

[cit22] Ma N., Li Y., Ren H., Xu H., Li Z., Zhang X. (2010). Polym. Chem..

[cit23] Li J., Huo M., Wang J., Zhou J., Mohammad J. M., Zhang Y., Zhu Q., Waddad A. Y., Zhang Q. (2012). Biomaterials.

[cit24] Kim H., Kim S., Park C., Lee H., Park H. J., Kim C. (2010). Adv. Mater..

[cit25] Wang C., Li Z., Cao D., Zhao Y. L., Gaines J. W., Bozdemir O. A., Ambrogio M. W., Frasconi M., Botros Y. Y., Zink J. I., Stoddart J. F. (2012). Angew. Chem., Int. Ed..

[cit26] Sun L., Liu W., Dong C. M. (2011). Chem. Commun..

[cit27] Šečkutė J., Devaraj N. K. (2013). Curr. Opin. Chem. Biol..

[cit28] Kurra Y., Odoi K. A., Lee Y. J., Yang Y., Lu T., Wheeler S. E., Torres-Kolbus J., Deiters A., Liu W. R. (2014). Bioconjugate Chem..

[cit29] Schoch J., Wiessler M., Jäschke A. (2010). J. Am. Chem. Soc..

[cit30] Jain S., Neumann K., Zhang Y., Geng J., Bradley M. (2016). Macromolecules.

[cit31] Wijnen J. W., Zavarise S., Engberts J. B. F. N., Charton M. (1996). J. Org. Chem..

[cit32] Lee B. F., Kade M. J., Chute J. A., Gupta N., Campos L. M., Fredrickson G. H., Kramer E. J., Lynd N. A., Hawker C. J. (2011). J. Polym. Sci., Part A: Polym. Chem..

[cit33] Karver M. R., Weissleder R., Hilderbrand S. A. (2011). Bioconjugate Chem..

[cit34] Zhang S., Liu X., Bawa-Khalfe T., Lu L. S., Lyu Y. L., Liu L. F., Yeh E. T. H. (2012). Nat. Med..

[cit35] Denard B., Seemann J., Chen Q., Gay A., Huang H., Chen Y., Ye J. (2011). Cell Host Microbe.

[cit36] Yang H., Wang Q., Huang S., Xiao A., Li F., Gan L., Yang X. (2016). ACS Appl. Mater. Interfaces.

[cit37] Du J. Z., Du X. J., Mao C. Q., Wang J. (2011). J. Am. Chem. Soc..

