# Supplementary material for: Nanoparticle “switch-on” by tetrazine triggering
Source: Chem Commun (Camb). 2016 Aug 19;52(75):11223–6. doi: 10.1039/c6cc05118a (PMC5048444; doi:10.1039/c6cc05118a)
Supplement: Supplementary file 1 [file CC-052-C6CC05118A-s001.pdf]

Electronic Supplementary Material (ESI) for ChemComm.
This journal is © The Royal Society of Chemistry 2016

Supplementary Information for

Nanoparticle "Switch-on" by Tetrazine Triggering

Kevin Neumann†, Sarthak Jain†, Jin Geng*, Mark Bradley*

List of Contents:

S1. General Information

S2. Synthesis of Tetrazine

S3. Polymer and Synthesis

S4. Preparation of vesicles

S5. Measurement of critical micellisation concentration (CMC)

S6. Evaluation of PC3 cell viability

S7. DOX release study from nanoparticles in PC3 Cells

S8. References

S1. General Information

Potassium (Acros) andFluorescein thiocyanate (Sigma Aldrich) were used as received. Allyl glycidyl ether (Sigma Aldrich) was distilled under vacuum. Naphthalene (Sigma Aldrich) was purified by recrystallization from hexane. Methoxypolyethylene glycol (M_n = 2000 g mol^{-1},

---

School of Chemistry, University of Edinburgh, David Brewster Road, Edinburgh, EH9 3FJ, United Kingdom. †These authors contributed equally to this work. *email: jin.geng@ed.ac.uk; mark.bradley@ed.ac.uk.

Sigma Aldrich) was dried by freeze-drying. Reactions, which required oxygen and moisture free conditions, were carried out with using Schlenk techniques under a nitrogen atmosphere.

^{1}H and ^{13}C nuclear magnetic resonance spectra were recorded on a Bruker AVA500 spectrometer (500 and 125 MHz respectively) at 298 K in deuterated solvents. Residual protic solvent ($CDCl_3$) signal was used as a reference (δ_h = 7.26 ppm, δ_C = 77.0 ppm), CHD_2OD (δ_H = 3.30 ppm, δ_C = 49.0 ppm), CHD_2SOCD_3 (δ_H = 2.50 ppm, δ_C = 39.5 ppm).

Coupling constants were measured in Hertz (Hz). Chromatographic purifications were carried out on silica gel 60-120 mesh. Analytical thin layer chromatography was performed on silica gel F254 (Merck). Low Resolution Mass Spectra were obtained using a Hewlett Packard LCMS 110 ChemStation with a G1946B mass detector. Reverse phase analytical HPLC (RP HPLC) was performed using an Agilent 1100 Chemstation on a Kinetex 5uXB-C18 (50 × 4 × 60 mm), and compounds were detected using an evaporative light scattering detector (ELSD) (Polymer Lab PL-ELS 1000 with simultaneous detection at 220, 254, 260, 282, and 495 nm). All solvents used were HPLC grade. Method A: eluent A: water and formic acid (0.1 %); eluent B: acetonitrile, formic acid (0.1%) (A = 95% 5 min, 95% to 5% over 10 min). Method B: eluent A: water and formic acid (0.1 %); eluent B: MeOH, formic acid (0.1%) (A = 95% 5 min, 95% to 5% over 10 min). Infrared absorption spectra were recorded on a SHIMADZU Iraffinity-1 CE FTIR spectrometer. The number of repeating units in a polymer were determined by ^{1}H-NMR via integration of the initiator signal and integration of the allyl moiety and was used to calculate the molecular weight of the polymers. TEM images were obtained by JEOL JEM-1400 Plus TEM. Representative images were collected on a GATAN OneView camera. Polymers were analysed by gel permeation chromatography (GPC) using two PLgel MIXED-C columns (200 - 2,000,000 g mol^{-1}, 5 mm) using *N, N*-dimethylformamide (DMF) with 0.1M LiBr at 60 ºC at 1 mL min^{-1} as the eluent. The GPC was calibrated with PMMA as standards. DLS (dynamic light scattering) measurements were carried out on a Malvern Zetasizer NanoZS at 25 ºC. Flow cytometry was performed on a BD FACSCanto™ II system. Propidium iodide (PI) (1 µg ml^{-1}) was added to the cells (5 mins in

the dark) after harvesting with trypsin. Each cell experiment was repeated for at least in triplicated.

Cell Analysis 40,000 PC3 cells were seeded in 48 flat-bottomed well plate. After 24 hours, cells were incubated with 20 □M of CellTracker™ Green (5-chloromethylfluorescein diacetate) in the dark. After washing with cell media, cells were incubated with nanoparticles loaded various concentrations of DOX. 50 µM tetrazine was used for triggering, with a Zeiss Axiovert 200M used to take continues images in each well every 1 hour for 72 hours at 37 °C with 5% CO_2.

S2. Synthesis of Tetrazine

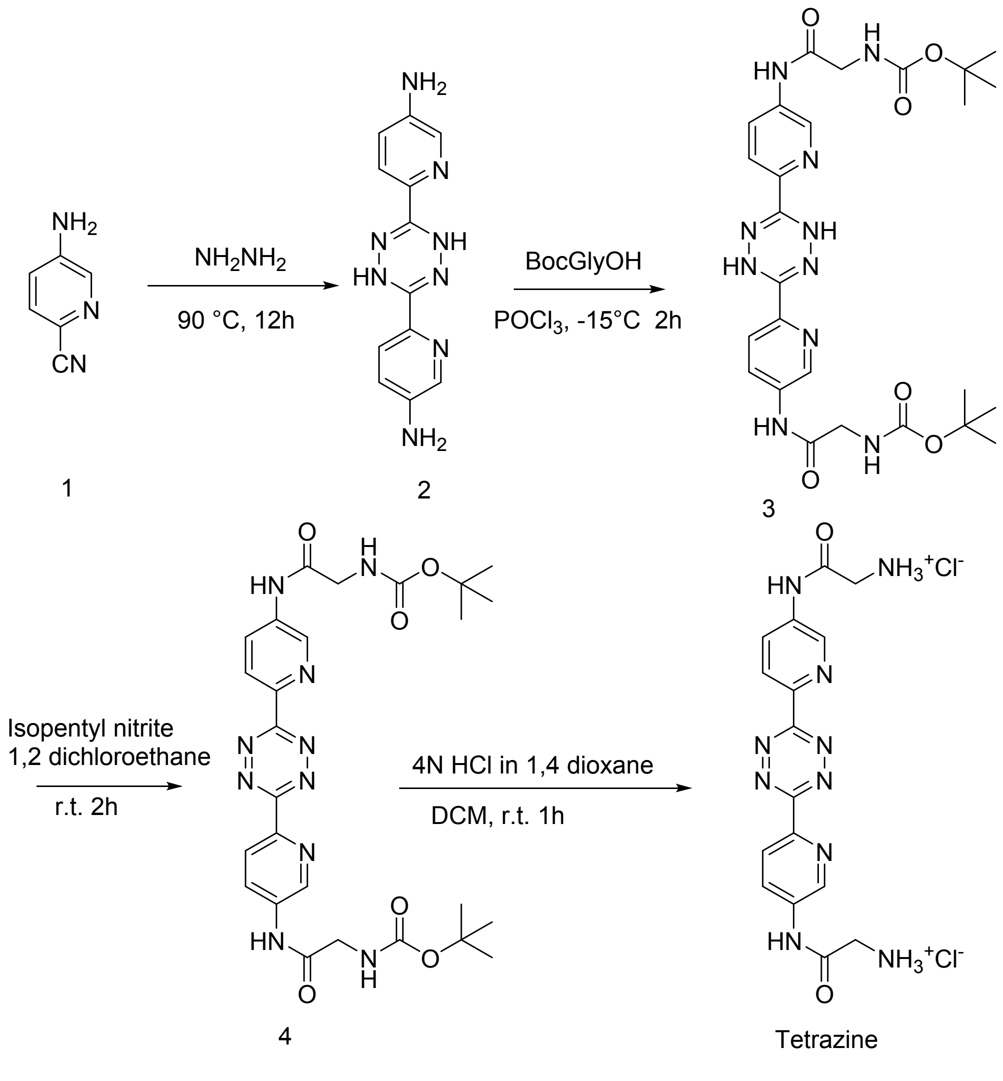

Synthesis of 2[1] 5-amino-2-cyanopyridine 1 (5.0 g, 42.0 mmol) in 64% aqueous hydrazine (10 ml, 322 mmol) was heated for 12 h to 90ºC behind a blast shield. The mixture was allowed to cool to r.t., the orange precipitate was isolated by filtration, washed with cold water and cold ethanol and dried under vacuum to afford the desired compound as an orange solid (2.9 g, 27% yield). HPLC (Method A) t_R 2.8 mins; FTIR 3280, 1575, 1385, 1273; ^{1}H NMR (500 MHz, DMSO-d_6) δ 8.40 (s, 2H), 7.92 (d, J = 2.5 Hz, 2H), 7.63 (d, J = 8.6 Hz, 2H), 6.99 (dd, J = 8.6, 2.5 Hz, 2H), 5.84 (s, 4H); ^{13}C NMR (126 MHz, DMSO-d_6) δ 147.54, 146.97, 135.05, 134.51, 122.05, 120.80; HRMS (m/z): $[M + H]^+$ calcd. for $C_{12}H_{13}N_8$, 269.12577; found, 269.12770.

Synthesis of 3 To a cold solution (-15 ºC) of N-(tert-butoxycarbonyl)glycine (0.9 g, 5.5 mmol) and 9 (0.7 g, 2.6 mmol) in anhydrous pyridine (16 mL), phosphoryl chloride (525 µL, 5.5 mmol) was added drop wise under N_2. The mixture was stirred for 2 hours at -15 ºC and then allowed to warm to room temperature and was stirred for an additional hour. The reaction mixture was quenched with water (1 mL). The solvents were evaporated under reduced pressure. The crude solid was dissolved in methanol, concentrated onto silica gel and the product was purified by chromatography silica gel (eluting with 0% to 10% methanol in dichloromethane with 1% TEA) as an orange solid (0.2 g, 13%). HPLC (Method A) t_R 5.4 mins; FTIR 3327, 1681, 1519, 1489, 1365; ^{1}H NMR (500 MHz, DMSO-d_6) δ 10.40 (s, 2H), 8.84 (s, 2H), 8.82 (d, J = 2.4 Hz, 2H), 8.16 (dd, J = 8.7, 2.5 Hz, 2H), 7.94 (d, J = 8.7 Hz, 2H), 7.13 (t, J = 6.1 Hz, 2H), 3.79 (d, J = 6.1 Hz, 4H), 1.41 (s, 18H). HRMS (m/z): $[M + Na]^+$ calcd. for $C_{26}H_{34}N_{10}O_6Na$, 605.25550; found, 605.25260.

Synthesis of 4 To a stirred solution of 10 (0.2 g, 0.3 mmol) in 1,2-Dicholoroethane (15 ml) Isopentyl nitrite (137 µl, 1.0 mmol) was added dropwise. After 2 hours the solvents were evaporated. Column chromatography on silica gel (eluting with 0% to 2% methanol in dichloromethane) afforded 4 as a pink solid (0.18 g, 90%). HPLC (Method A) t_R 4.9 mins; FTIR 3327, 1676, 1519, 1288; ^{1}H NMR (500 MHz, DMSO-d_6) δ 10.59 (s, 2H), 9.05 (d, J =

2.5 Hz, 2H), 8.62 (d, J = 8.7 Hz, 2H), 8.42 (dd, J = 8.8, 2.5 Hz, 2H), 3.84 (d, J = 6.1 Hz, 4H), 1.42 (s, 18H); ^{13}C NMR (126 MHz, DMSO-d6) δ 169.99, 163.00, 156.46, 144.63, 141.74, 138.59, 126.78, 125.23, 78.69, 44.42, 28.70; HRMS (m/z): $[M + H]^+$ calcd. for $C_{26}H_{33}N_{10}O_6$, 581.25791; found, 581.25780.

Synthesis of Tetrazine To a stirred solution of 4 (0.18 g, 0.3 mmol) in dichloromethane (14 ml) was added a 4N HCl solution in dioxane (8 ml) and the reaction mixture was allowed to stir for 1 hour at r.t., after which complete consumption of the starting material was observed by LC-MS and TLC analysis. The solvent was removed and Et_2O (30 ml) was added and the precipitate was recovered by filtration (0.14 g, quantitative). HPLC (Method B) t_R 0.8 mins; FTIR 2968, 1606, 1568, 1381; ^1H NMR (500 MHz, DMSO-d_6) δ 11.27 (s, 2H), 9.12 (d, J = 2.5 Hz, 2H), 8.68 (d, J = 8.7 Hz, 2H), 8.42 (dd, J = 8.7, 2.5 Hz, 2H), 8.24 (br, 6H), 3.94 (d, J = 5.7 Hz, 4H); ^{13}C NMR (126 MHz, DMSO-d_6) δ 166.58, 163.01, 145.32, 141.79, 137.91, 127.12, 125.42, 41.78; HRMS (m/z): $[M +H]^+$ calcd. for $C_{16}H_{17}N_{10}O_2$, 381.15305; found, 381.15420.

S3. Polymer synthesis

PEG-*b*-PAGE[2] Naphthalene (12.8 mg, 0.1 mmol) was dissolved in dry THF (2.5 mL) and potassium (4 mg, 0.1 mmol) was added and the suspension was stirred for 1 hour. The solution was added to methoxypolyethylene glycol (2000 g mol^{-1}) (200 mg, 0.1 mmol). After 10 min allyl glycidyl ether (430 µl, 3.7 mmol) was added. The reaction was stirred for 20 h at 30 °C, before being quenched with methanol (1 ml). The polymer was dissolved in dichloromethane (50 ml), washed twice with water (10 ml) and the organic phase dried over sodium sulfate before removal of the solvents under reduced pressure, to give the polymer as a colorless oil.

GPC (DMF): M_n 3130 g mol^{-1}, PDI 1.03

^{1}H-NMR (500 MHz, $CDCl_3$): δ 5.83 – 5.91 (m, 32H, CH_2-O-CH_2-C$\underline{H}$=CH_2), 5.12 – 5.29 (m, 64H, CH_2-O-CH_2-CH=C$\underline{H_2}$), 3.95 – 4.03 (m, 64H, CH_2-O-C$\underline{H_2}$-CH=CH_2), 3.50 – 3.75 (m, 205H, -C$\underline{H_2}$-C$\underline{H}$(C$\underline{H_2}$-O-CH_2-CH=CH_2)-O-) and -C$\underline{H_2}$-C$\underline{H_2}$-O-).

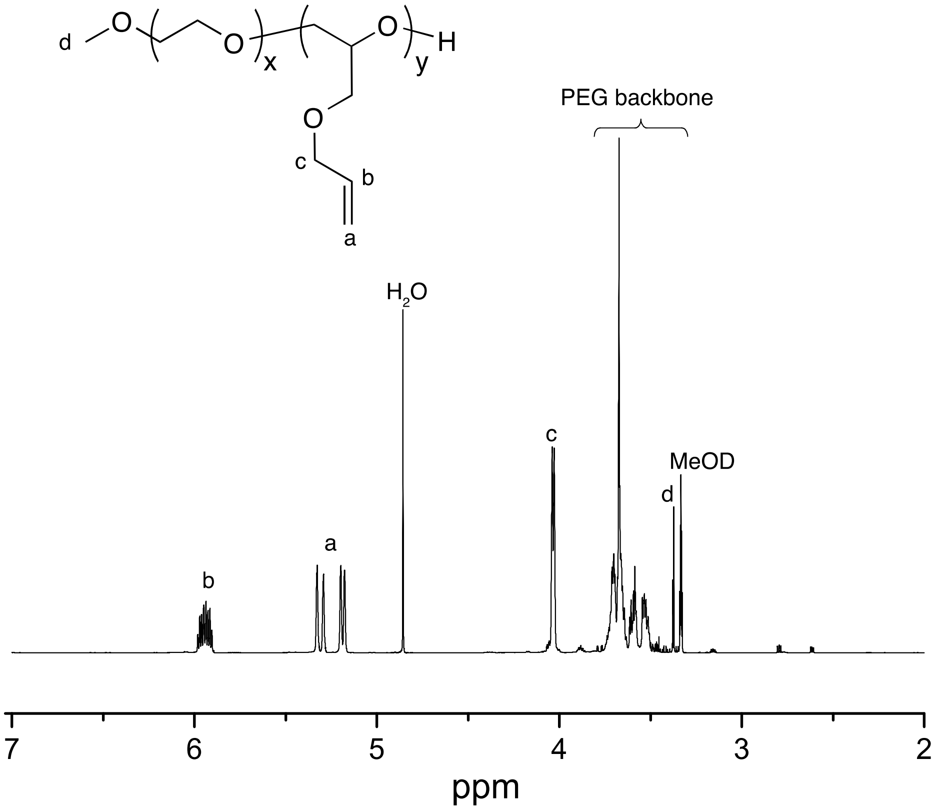

Figure S1. Proton NMR spectrum of **PEG-*b*-PAGE**.

Fluorescein labeled Polymer (PEG-*b*-PAGE-FITC)

Flourescein thiocyanate (FITC) (5 mg, 12.5 µmol, 2 equiv.) and PEG-*b*-PAGE (50 mg, 6.75 µmol, 1 equiv.) were dissolved in DMF (3 ml) and stirred in the dark for 24 h. After precipitating with water (2 ml) the polymer was separated from the water by centrifugation (3000 rpm, 5 min). After freeze drying the polymer was obtained as a yellow solid (48 mg, 90 %).

GPC (DMF): M_n 3996 g mol^{-1}; PDI 1.26.

^{1}H-NMR (500 MHz, $CDCl_3$): δ 7.07 – 7.14 (m, 2H, FITC), 6.73 – 6.78 (m, 2H, FITC), 6.53 – 6.68 (m, 4H, FITC), 6.38 – 6.44 (m, 1H, FITC), 5.83 – 5.91 (m, 32H, CH_2-O-CH_2-C$\underline{H}$=CH_2),

5.12 – 5.29 (m, 64H, CH_2-O-CH_2-CH=C$\underline{H_2}$), 3.95 – 4.03 (m, 64H, CH_2-O-C$\underline{H_2}$-CH=CH_2), 3.50 – 3.75 (m, 205H, -C$\underline{H_2}$-C$\underline{H}$(C$\underline{H_2}$-O-CH_2-CH=CH_2)-O-) and -C$\underline{H_2}$-C$\underline{H_2}$-O-).

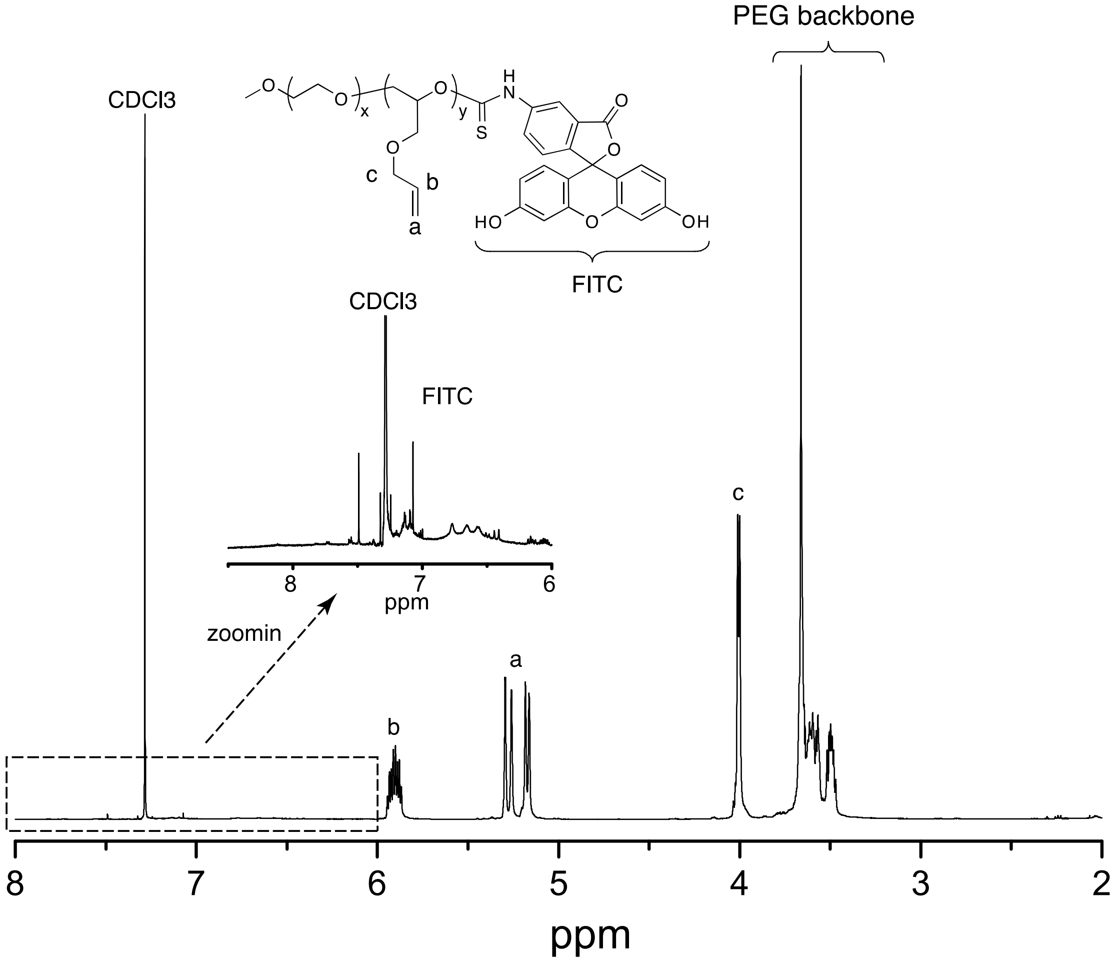

Figure S2. Proton NMR spectrum of **PEG-*b*-PAGE-FITC**.

S4. Preparation of vesicles

PEG-*b*-PAGE was dissolved in DMF (30 mg ml^{-1}) and precipitated drop-wise with water (3 mg ml^{-1} of polymer). For encapsulation studies PEG-*b*-PAGE in DMF was precipitated with water (final concentration 3 mg ml^{-1}) containing the desired cargo (DOX from 5 μM to 20 μM). The nanoparticles were purified by centrifugation (3000 rev min^{-1}) for 20 min. The supernatant was removed and the procedure was repeated twice before the nanoparticles were finally re-dissolved in the desired milieu.

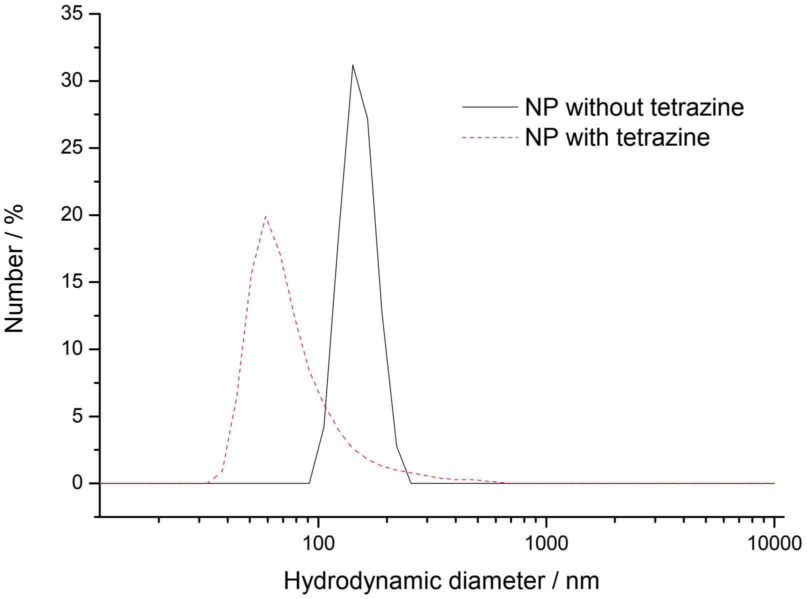

Figure S3. DLS data for **PEG-*b*-PAGE** nanoparticle before and after reaction with tetrazine. Nanoparticles were prepared at concentration of 3 mg ml^{-1} in water and incubated with tetrazine at 37 ºC for 24 hours. [Tetrazine]: [allyl ether] = 4 : 1.

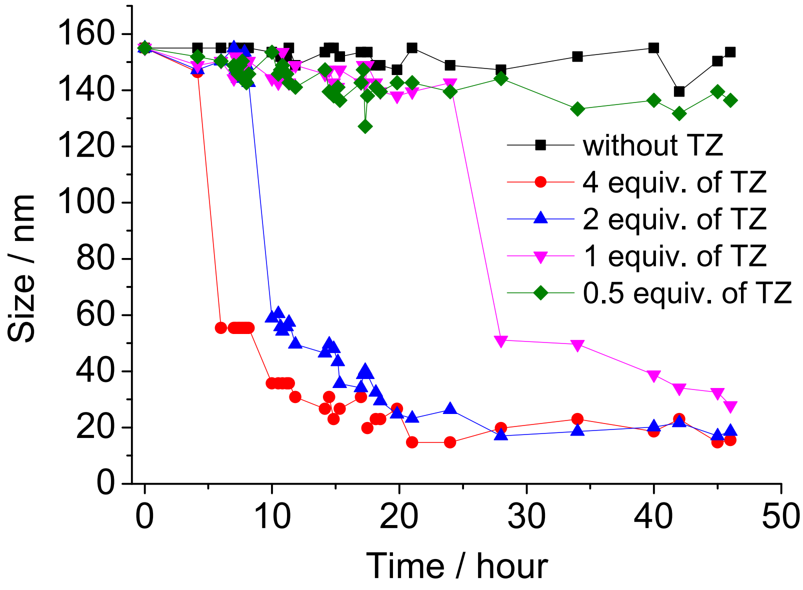

Figure S4. Size change of vesicles in the presence of different equivalents of tetrazine to allyl groups of polymer.

S5. Measurement of critical micellisation concentration (CMC)

An aqueous solution of benzyl acetone (BZA) (0.06 mmol l^{-1}) was prepared. PEG-*b*-PAGE solutions at various concentrations were prepared using the BZA solution. Absorbance of prepared samples was measured at 312 nm.

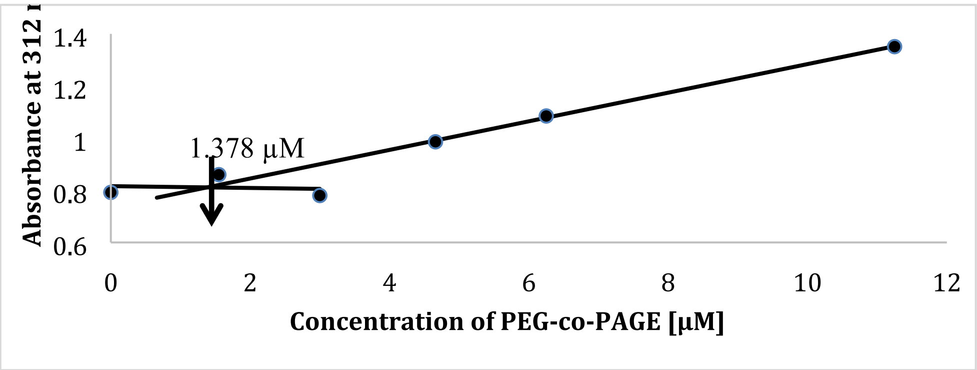

Figure S5. Absorbance of BZA as a function of PEG-*b*-PAGE at 312 nm (CMC = 1.378 µM).

S6. Evaluation of PC3 cell viability[4]

Live/dead staining was performed with FDA (Fluorescein Diacetate) and PI (Propidium iodide).

PC3 cells were seeded in 48 well plate for 24 hours. Desired concentrations of tetrazine, NP's, NP's loaded with Dox and NP loaded with DOX and trigger tetrazine were incubated for 72 hours. After 72 hours, cells were washed with serum free DMEM media (two times) and then stained solution (FDA/PI) was added. The cells were incubated for 10 min at 37 °C in the dark before obtaining fluorescent images using a Zeiss Axiovert 200M microscope.

The staining solution was prepared using by mixing 8 µl of FDA (5 mg ml^{-1} in acetone), 50 µl PI (2 mg ml^{-1} in PBS) with 5 ml serum free DMEM.

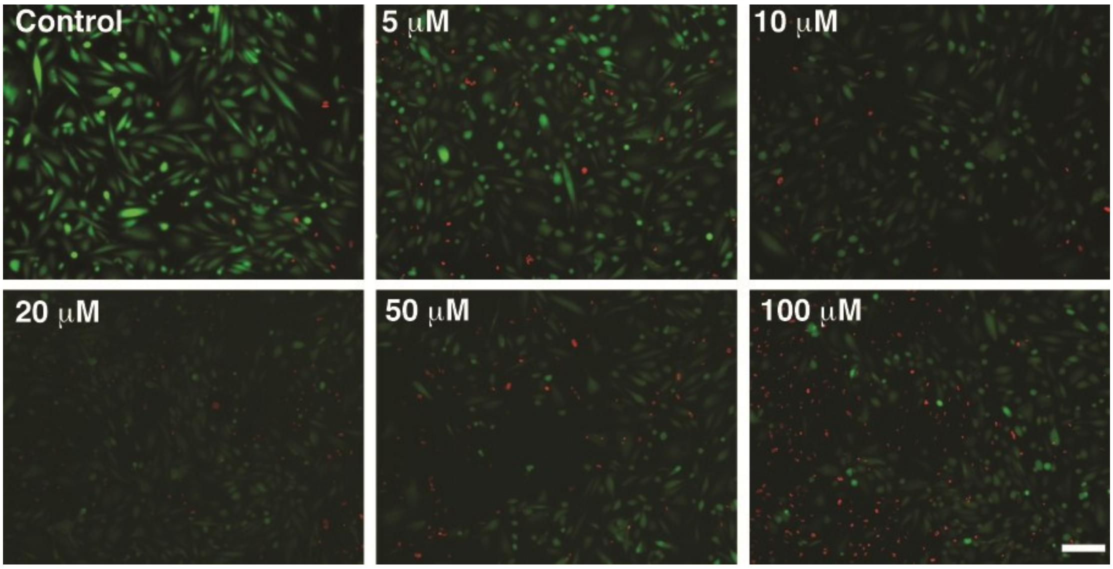

Figure S6. PC3 cells incubation with nanoparticles (formed by PEG-*b*-PAGE) with the concentration of from 5 μM to 100 μM of PEG-*b*-PAGE for 72 hours. Green fluorescence due to FDA staining (FITC Channel λ_{ex} = 447-494 nm; λ_{em} = 500-554 nm). Red fluorescence due to PI staining (TRITC Channel λ_{ex} = 527-563 nm; λ_{em} = 570-650 nm).

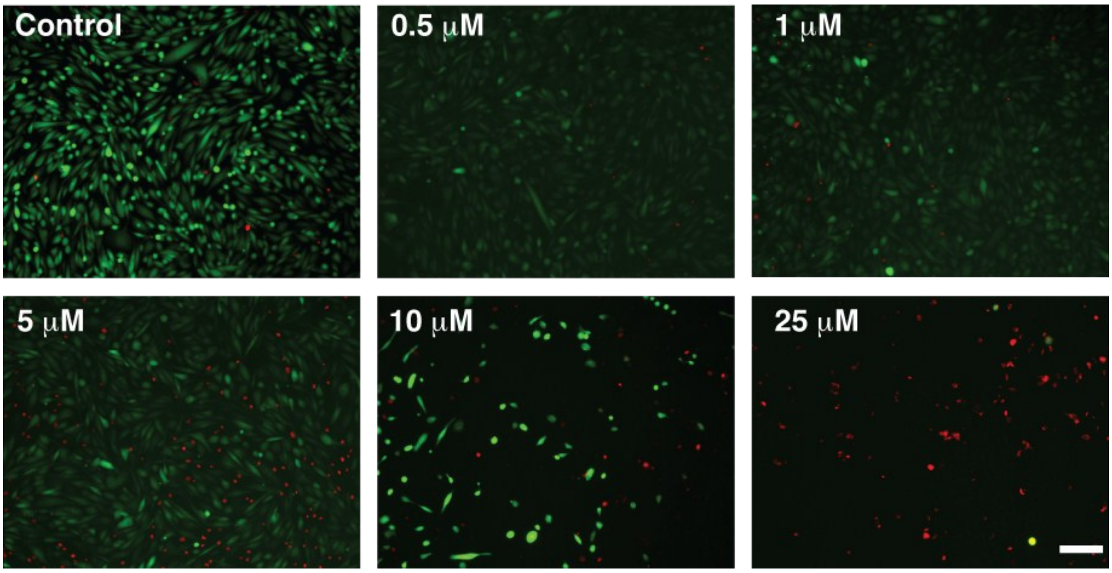

Figure S7. PC3 cells incubation with DOX at 0.5 μM to 25 μM for 72 hours. Green fluorescence due to FDA staining (FITC Channel λ_{ex} = 447-494 nm; λ_{em} = 500-554 nm). Red fluorescence due to PI staining (TRITC Channel λ_{ex} = 527-563 nm; λ_{em} = 570-650 nm).

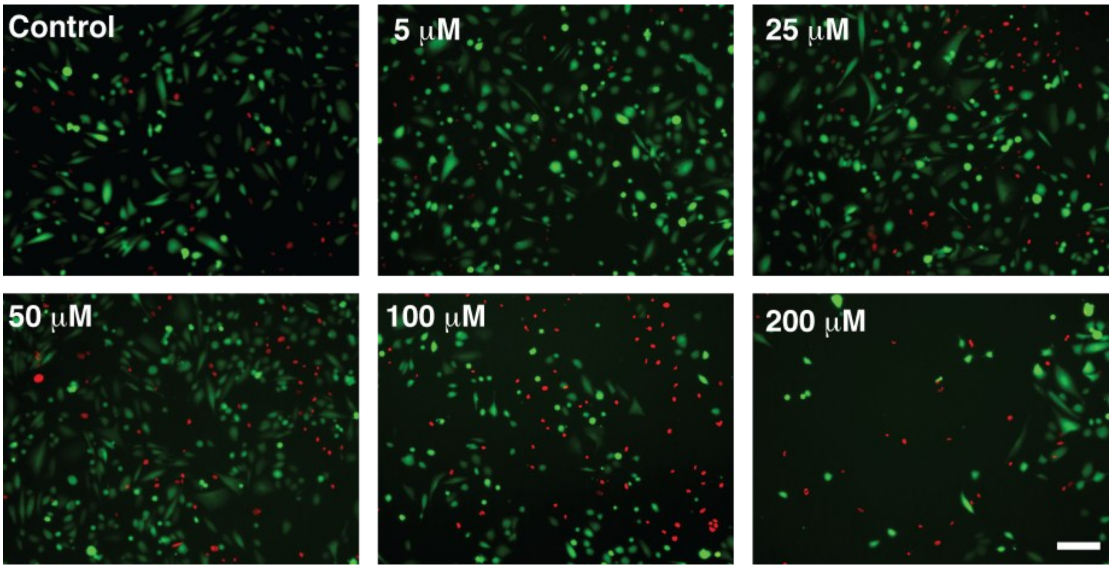

Figure S8. PC3 cells incubation with tetrazine at 5 μM to 200 μM for 72 hours. Green fluorescence due to FDA staining (FITC Channel λ_{ex} = 447-494 nm; λ_{em} = 500-554 nm). Red fluorescence due to PI staining (TRITC Channel λ_{ex} = 527-563 nm; λ_{em} = 570-650 nm).

S7. DOX release study from nanoparticles in PC3 Cells

Determination of encapsulated amount of Doxorubicin

Nanoparticles were prepared with different concentrations of Doxorubicin (1 μM, 2 μM, 20 μM, 50 μM and 100 μM). Fluorescence emission of the samples was measured and calibrated from a calibration curve prepared using a series of samples of known DOX concentrations.

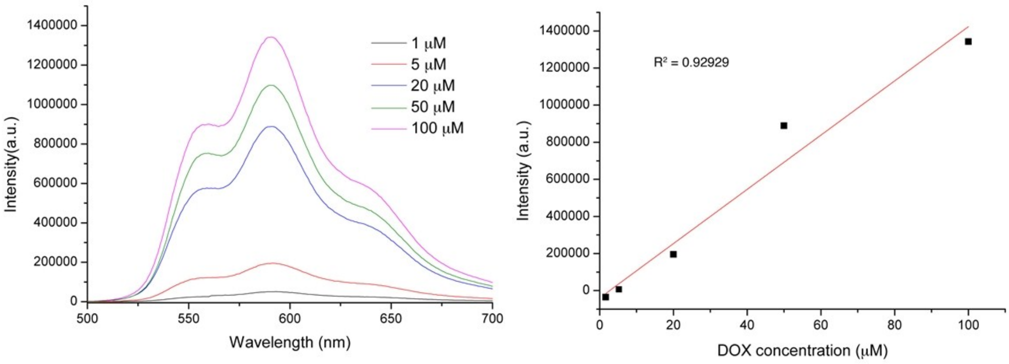

Figure S9. Fluorescence emission spectra (excited at 470 nm for DOX) at various

concentrations in water at 25 ºC. A calculation curve was created using intensity at λ_{em} = 591 nm over the different concentration of DOX. The concentration of DOX loaded in the nanoparticles were calculated accordingly. The encapsulation efficiency (%) value was calculated according to the following equations:

$$Encapsulation\ Efficiency\ (\%) = \frac{Weight\ of\ DOX\ in\ NPs}{Weight\ of\ DOX\ in\ feed} \times 100\%$$

Release study of DOX-loaded nanoparticles The nanoparticles loaded with DOX were prepared by dissolving **PEG-*b*-PAGE** in DMF and then treated with a solution of DOX in water. To confirm the total amount of DOX encapsulated, NPs were isolated by centrifugation and then dissolved in DMF and quantified by fluorescence.

DOX NPs (3 mg ml^{-1}) was loaded into 20 kDa MWCO dialysis tubing and dialysed against 30 mL of phosphate buffered saline (PBS, pH 7.4) in the dark at 37 ºC. At specified time points, 100 µl of the dialysis buffer was collected (replaced with equal volume of fresh PBS) and the concentrations of DOX present in the dialysate determined (λ_{ex} 480nm and λ_{em} 530nm). Tetrazine (40 mg, 0.08 mmol, 4 equiv. to the allyl ether units) was introduced into the above solution after 4 hours and the release profile was monitored.

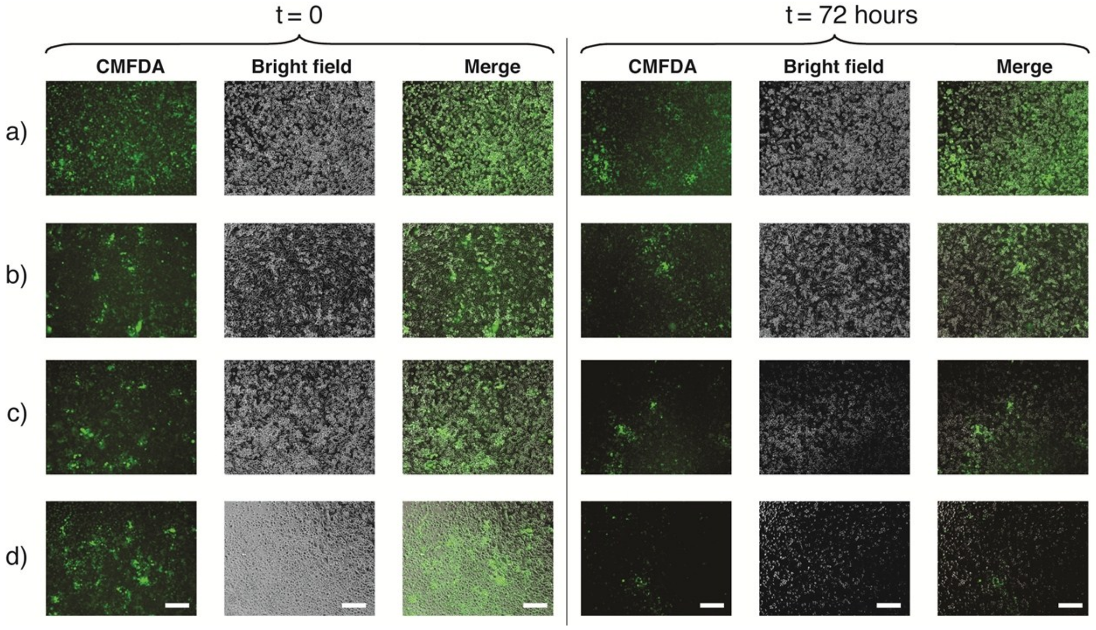

Figure S10. Microscope images of PC3 cells with DOX-loaded NPs (17 µg ml^{-1}) in the present of tetrazine (50 µM) at t = 0 and t = 72 hours: a) Empty NPs; b) NPs loaded with 1 µm DOX; c) NPs loaded with 6 µM DOX; d) NPs loaded with 18 µM DOX.

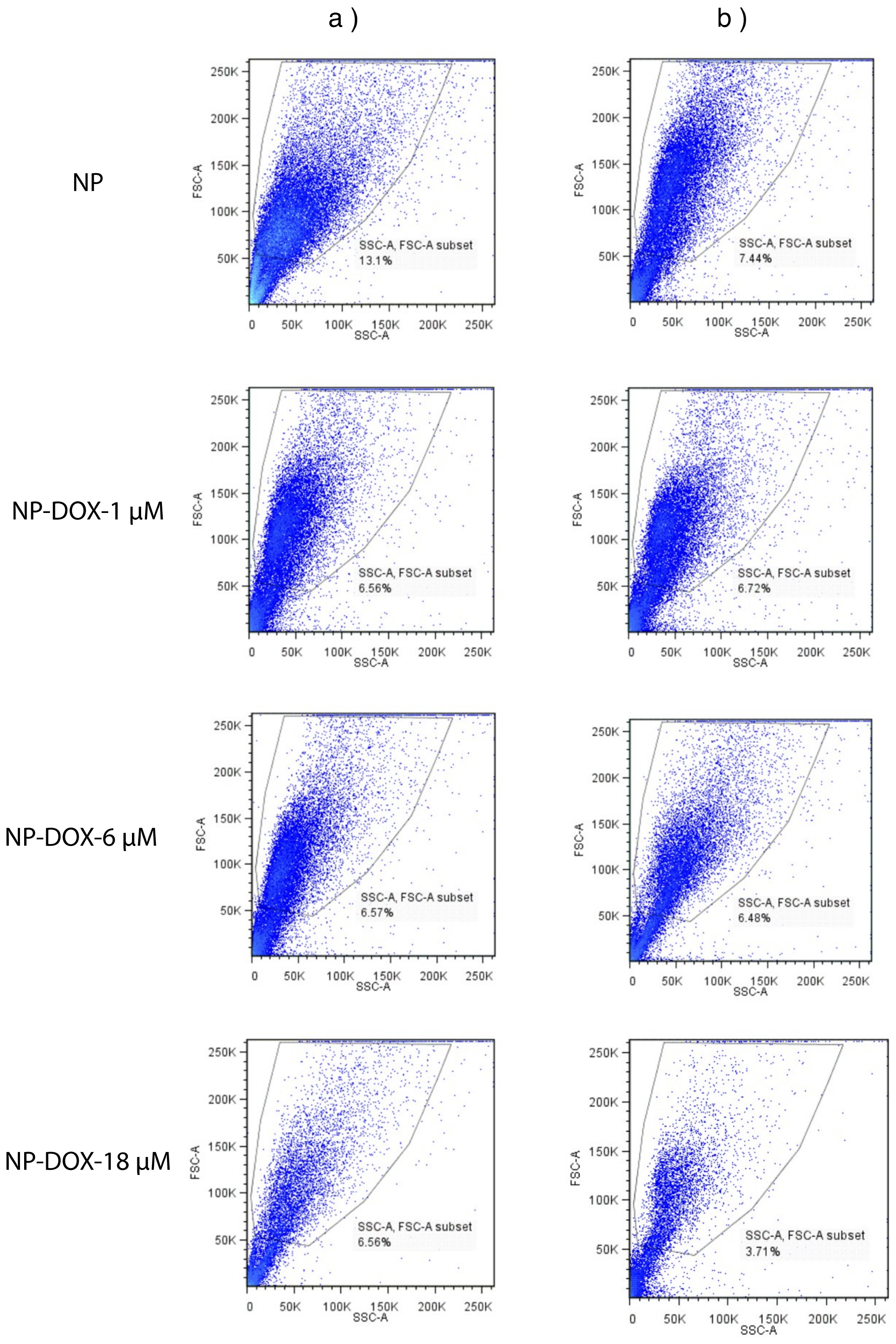

Figure S11. Forward versus Side scatter profiles that were used to gate intact cellular materials (remove cell debris and dead cells). PC3 cells incubated with nanoparticles (17 µg

ml^{-1}) a) in the absent of tetrazine; b) in the present of tetrazine (50 μM) for t = 72 hours at 37 ºC with 5% CO_2.

S8. References

1. Asselin, C. M. *et al.* Synthesis and metallation of ferrocenylimines derived from ligating diaminoheteroarenes. *J Chem Soc Dalton* **0,** 3765–3772 (1997).
2. Lee, B. F. *et al.* Poly(allyl glycidyl ether)-A versatile and functional polyether platform. *J Polym Sci Pol Chem* **49,** 4498–4504 (2011).
3. Peppas, N. A. & Sahlin, J. J. A simple equation for the description of solute release. III. Coupling of diffusion and relaxation. *Int J Pharm* **57,** 169–172 (1989).
4. Jones, K. H. & Senft, J. A. An improved method to determine cell viability by simultaneous staining with fluorescein diacetate-propidium iodide. *J Histochem Cytochem* **33,** 77–79 (1985).
